# Physiological Effects, Molecular Regulation, and Adaptive Strategies of Heat Stress in Fish Under Global Warming

**DOI:** 10.3390/ani16162573

**Published:** 2026-08-18

**Authors:** Jiqin Huang, Hongying Ma, Hui Yang, Jianlu Zhang

**Affiliations:** 1 Shaanxi Key Laboratory of Qinling Ecological Security, Shaanxi Institute of Zoology, Xi’an 710032, China; 2 College of Animal Science and Technology, Yangzhou University, Yangzhou 225009, China; 3 College of Urban and Environmental Sciences, Northwest University, Xi’an 710127, China

**Keywords:** aquaculture, heat tolerance, oxidative stress, metabolic reprogramming, intestinal barrier, inflammatory signaling, thermal acclimation, climate resilience

## Abstract

Heat stress is one of the most prevalent and unavoidable environmental stressors in modern aquaculture and natural fishery ecosystems. With the intensification of global warming, the frequency, intensity, and duration of extreme heat events have increased substantially, progressively reducing the thermal safety margin of fish. As ectothermic vertebrates, fish rely heavily on ambient water temperature to maintain physiological functions and metabolic homeostasis. Elevated temperatures can induce profound metabolic reprogramming, characterized by suppressed protein deposition, enhanced lipid mobilization, and altered glucose metabolism, while simultaneously causing structural damage and functional dysfunction in critical organs and tissues such as the liver, intestine, gills, and reproductive system. Heat stress also activates the stress axis and oxidative stress responses, often leading to immunosuppression and disruption of antioxidant defenses, ultimately impairing growth performance, behavior, reproduction, and survival. This review focuses on fish and summarizes the major physiological injuries, molecular regulatory pathways, and adaptive strategies associated with heat stress under global warming. Comparative evidence from crustaceans and molluscs is used only where it helps clarify shared or divergent responses among aquatic ectotherms.

## Simple Summary

Global warming and frequent heatwaves have made heat stress a major challenge for fish in aquaculture and natural ecosystems. High temperatures can disrupt energy metabolism, damage important organs, weaken immunity, and reduce growth and survival. This review summarizes the physiological and molecular responses of fish to heat stress, including oxidative stress, inflammation, and metabolic regulation pathways. It also discusses potential adaptive strategies, such as temperature acclimation and nutritional regulation, to improve thermal tolerance and support sustainable aquaculture and fishery resource conservation under climate change.

## 1. Introduction

Heat stress refers to a series of abnormal physiological and behavioral responses that occur when environmental water temperature exceeds the thermal tolerance range of fish, and it has become one of the most prevalent and unavoidable environmental stressors in aquaculture and natural fishery ecosystems [1]. Typical symptoms include surfacing behavior, reduced or ceased feeding, increased opercular movement, abnormal mucus secretion, and suppression of immune function, often accompanied by body surface redness and inflammatory congestion. In severe cases, both acute and chronic heat exposure may ultimately lead to mortality. Elevated temperature not only directly reduces dissolved oxygen levels in water and induces hypoxic stress, but also disrupts metabolic homeostasis, enhances oxidative stress, and causes multi-organ damage, thereby posing serious threats to aquaculture production and fisheries’ resource sustainability.

Global climate change has profoundly altered aquatic ecosystems worldwide. Rising sea surface temperatures, increased frequency and duration of marine heatwaves, intensified thermal stratification, and progressive deoxygenation are continuously reshaping the ecological and physiological environments experienced by aquatic organisms. These changes compress the thermal safety margins of fish, alter species distribution patterns and energy allocation strategies, and increase the risks of pathogen outbreaks and ecosystem instability. In both marine and freshwater environments, prolonged heat exposure has become a major environmental factor affecting fish growth, reproduction, immunity, and survival. In aquaculture systems, especially during summer heatwaves, pond waters can rapidly warm beyond the upper thermal tolerance limit of cultured species, often accompanied by hypoxia and deterioration of water quality. Similarly, marine cage aquaculture, particularly Atlantic salmon farming in Norway, Chile, Canada, and Tasmania, has experienced severe economic losses caused by heat-induced deoxygenation and disease outbreaks. These events highlight the increasing vulnerability of intensive aquaculture systems to global warming. High temperatures also accelerate the decomposition of residual feed and feces, increasing the accumulation of ammonia nitrogen and nitrite and further amplifying physiological stress in fish.

Thermal tolerance differs substantially among aquatic species. For example, feeding inhibition commonly occurs when temperatures exceed 33 °C in some cultured fish species, while temperatures above 35 °C may induce large-scale mortality [2]. Tilapia (*Oreochromis* spp.) are relatively heat-tolerant, but prolonged exposure to excessively high temperatures can still lead to respiratory distress, growth inhibition, immune suppression, and elevated mortality risk [3]. The optimal growth temperature of whiteleg shrimp (*Litopenaeus vannamei*) is 25–30 °C, whereas temperatures above 38 °C can cause molting disorders and immune dysfunction. In Chinese shrimp (*Fenneropenaeus chinensis*), survival declines markedly above 33 °C [4]. The optimal temperature range for Chinese mitten crab (*Eriocheir sinensis*) is 20–28 °C, and prolonged exposure to temperatures above 32 °C can trigger abnormal escape behavior, including rapid swimming, surface jumping, and attempts to avoid high-temperature zones, ultimately resulting in mass mortality [5].

**Figure 1:**
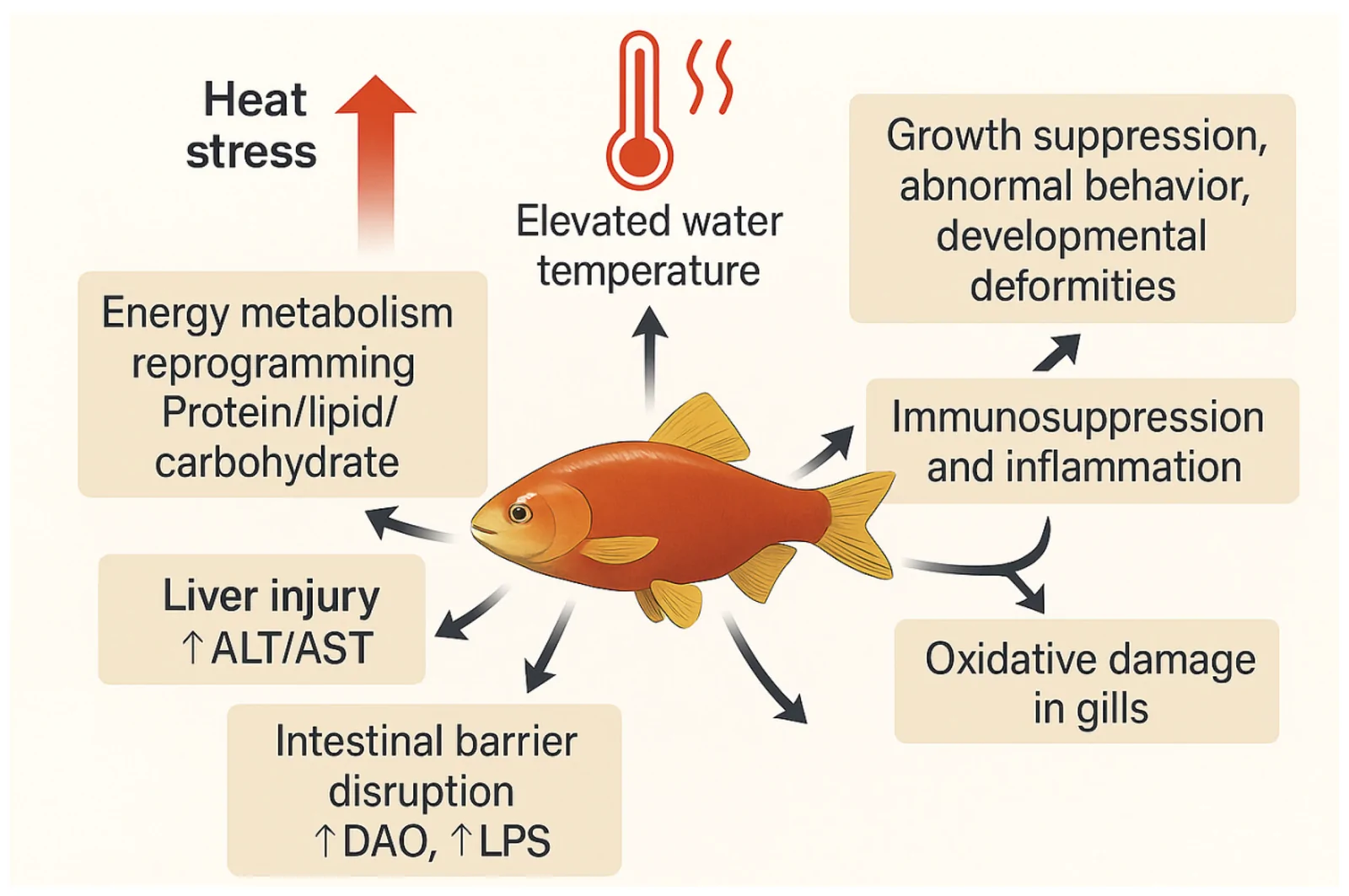
Schematic overview of physiological damage caused by heat stress in fish. ↑ indicates an increase or upregulation. ALT, alanine aminotransferase; AST, aspartate aminotransferase; DAO, diamine oxidase; LPS, lipopolysaccharide.

As typical poikilothermic vertebrates, fish lack effective internal thermoregulatory mechanisms, and many physiological processes are highly dependent on ambient water temperature. Under heat stress conditions, fish undergo a series of adaptive and pathological changes involving energy metabolism, endocrine regulation, physiological and biochemical parameters, and gene expression. Heat stress can induce metabolic reprogramming characterized by suppressed protein deposition, enhanced lipid mobilization, and altered glucose metabolism, while simultaneously causing structural damage and functional dysfunction in critical tissues and organs such as the liver, intestine, gills, and reproductive system. In addition, heat stress activates the stress axis and oxidative stress responses, resulting in immunosuppression and disruption of antioxidant defenses, which ultimately impair growth performance, behavior, and survival. The major physiological injuries induced by heat stress in fish are summarized schematically in Figure 1.

Recent multi-omics studies have shown that fish respond to heat stress through an intricate regulatory network centered on heat shock protein (HSP)-mediated molecular chaperone systems and multiple signaling pathways, including Nrf2-Keap1, FOXO, PI3K/Akt, MAPK, NF-kappa B, FXR/TGR5, and PPAR. Together, these pathways coordinate antioxidant defense, inflammatory regulation, apoptosis, and metabolic remodeling. In parallel, adaptive approaches such as thermal acclimation and nutritional interventions, including allicin, bile acids, appropriate dietary lipid levels, and other functional nutrients where supported by species-specific evidence, may improve heat tolerance and reduce tissue damage.

Accordingly, this review is organized around a sequential framework of “environmental heat exposure–physiological injury–molecular regulation–adaptive intervention.” Within this framework, heat stress is first considered as a climate-driven environmental challenge of direct relevance to aquaculture and fisheries. Its consequences are then examined from energy metabolic disturbance and tissue injury to immune dysfunction and oxidative imbalance, followed by a synthesis of the molecular pathways involved. Finally, practical response strategies, including environmental acclimation, nutritional regulation, selective breeding, and management interventions, are evaluated in terms of their potential to improve resilience under warming conditions. This integrative structure is intended to link physiological outcomes, signaling mechanisms, and intervention strategies into a coherent synthesis rather than treating them as separate topics.

Under global warming, this review systematically summarizes the physiological effects, molecular regulatory mechanisms, and adaptive strategies associated with heat stress in fish. It also discusses the implications of heat stress for aquaculture production and fishery resource management, with the aim of providing a theoretical basis for the development of heat-tolerant strains, the optimization of aquaculture practices, and the formulation of climate-adaptive fisheries management strategies.

The specific knowledge gap addressed by this review lies in the limited integration of organism-level heat injury, pathway-level molecular regulation, and application-oriented mitigation strategies in fish under global warming. The primary applied context is aquaculture, because cultured fish are directly exposed to increasingly frequent heatwaves and because mitigation measures such as water-quality control, thermal acclimation, nutritional regulation, selective breeding, and early-warning systems can be implemented in aquaculture systems. Wild fisheries are discussed only as an extension of the broader ecological and resource-management implications of heat stress, including habitat shifts, recruitment changes, disease risk, and population stability. Crustaceans and shellfish are included only as complementary comparative evidence when they help clarify conserved or divergent heat-stress responses among aquatic ectotherms.

## 2. Literature Search Strategy

Literature search strategy: relevant publications were identified mainly from Web of Science, PubMed, ScienceDirect, SpringerLink, Wiley Online Library, and Google Scholar. Search terms included combinations of “heat stress”, “thermal stress”, “high temperature”, “fish”, “aquatic animals”, “aquaculture”, “oxidative stress”, “immune response”, “energy metabolism”, “heat shock protein”, “Nrf2”, “MAPK”, “NF-kappa B”, “PI3K/Akt”, “PPAR”, “bile acid”, “thermal acclimation”, and “nutritional regulation”. Priority was given to peer-reviewed studies that reported physiological, biochemical, histological, transcriptomic, metabolomic, immune, or growth-related responses to elevated temperature in fish and closely related aquaculture species. Studies were excluded when they lacked clear temperature conditions, exposure duration, species information, or direct relevance to heat-stress mechanisms or mitigation. Review articles were used mainly to support conceptual framing, whereas experimental studies were used preferentially for mechanistic and species-specific evidence.

## 3. Effects of Heat Stress on the Physiological Functions of Fish

### 3.1. Effects of Heat Stress on Energy Metabolism in Fish

Heat stress exerts profound regulatory effects on energy metabolism in fish, particularly on protein, lipid, and carbohydrate metabolism. In general, elevated temperatures suppress protein deposition while promoting lipid mobilization, thereby shifting energy utilization toward lipids and carbohydrates. Evidence from fish studies indicates that acute or chronic heat exposure can alter amino acid metabolism, reduce protein retention, and impair growth-related metabolic balance [6,7,8]. Heat stress can also activate the hypothalamic–pituitary–interrenal (HPI) axis and stimulate cortisol secretion, which is considered one of the major endocrine responses to heat stress in fish. In several teleost species, acute heat stress has been reported to increase circulating cortisol levels, although the magnitude and duration of this response are highly species-specific and depend on stress intensity, exposure duration, developmental stage, and acclimation history. Elevated cortisol levels may promote protein catabolism in muscle and liver tissues, thereby contributing to reduced nitrogen retention, impaired growth performance, and metabolic imbalance during prolonged heat exposure [7,8,9].

Heat shock proteins (HSPs), which function as important molecular chaperones during cellular stress responses, play essential protective roles against heat-induced protein denaturation and aggregation. In Rohu (*Labeo rohita*), heat stress altered protein metabolism and significantly reduced blood glucose, blood lipid, and total protein levels, while simultaneously increasing the activities of glutamate pyruvate transaminase (GPT) and glutamate oxaloacetate transaminase (GOT), indicating liver dysfunction accompanied by anemia, ultimately leading to impaired growth and mortality [9]. Similarly, studies in razor clams (*Sinonovacula constricta*) demonstrated that high-temperature exposure significantly upregulated the expression of molecular chaperones such as HSP70 and HSP90, as well as endoplasmic reticulum stress-related proteins, thereby promoting the repair or degradation of misfolded proteins and maintaining protein homeostasis [10].

Heat stress also disrupts lipid metabolic homeostasis in fish, mainly through enhanced lipolysis, suppressed lipid synthesis, and membrane lipid remodeling. Elevated temperature can activate lipolytic enzymes such as hormone-sensitive lipase (HSL) and lipoprotein lipase (LPL), promoting triglyceride hydrolysis in adipose tissue and the liver to generate free fatty acids (FFA). For example, in rainbow trout (*Oncorhynchus mykiss*) exposed to 24 °C heat stress, serum FFA levels increased by approximately 60% compared with the control group, while hepatic HSL gene expression increased 2.1-fold, indicating a marked enhancement of lipid mobilization [11]. At the same time, high temperature inhibits the activities of key lipogenic enzymes, including acetyl-CoA carboxylase and fatty acid synthase, thereby reducing hepatic lipid synthesis capacity. In razor clams, heat stress also downregulated the expression of fatty acid synthesis-related genes such as FASN and Elovl2/5, suggesting that aquatic animals generally shift lipid metabolism toward energy mobilization under thermal stress conditions [12].

Carbohydrates are among the most rapidly mobilized energy substrates in fish and can be converted into ATP through glycolysis, the tricarboxylic acid (TCA) cycle, and mitochondrial oxidative phosphorylation. Heat stress frequently disrupts glucose homeostasis, typically manifesting as altered blood glucose levels during acute heat exposure and metabolic pathway remodeling under chronic heat stress [13,14]. In large yellow croaker (*Larimichthys crocea*), high temperature altered serum biochemical indices, indicating rapid metabolic adjustment under elevated temperature [15]. In juvenile Qingtian rice carp (*Cyprinus carpio*), heat stress significantly increased hepatic glucose and D-gluconic acid levels, suggesting enhanced gluconeogenesis and glycogenolysis to meet increased energy demands under high temperature [8]. In Pacific oyster (*Crassostrea gigas*), prolonged exposure to elevated temperature inhibited glycogen synthesis and mobilization, exacerbated energy imbalance during reproduction, and contributed to summer mortality [16]. These findings indicate that heat-stress-induced carbohydrate responses are species- and context-dependent and should not be generalized without considering exposure duration and life stage.

### 3.2. Damage of Heat Stress to Fish Tissues and Organs

The liver is the largest digestive gland and central metabolic organ in fish. It plays essential roles in glycogen storage, detoxification metabolism, and the clearance of pathogens, toxic compounds, and metabolic waste. Under heat stress conditions, hepatic metabolic efficiency may decline, which can result in toxin accumulation, aggravated liver injury, and reduced disease resistance. Elevated temperature can increase hepatocyte membrane permeability, leading to the release of intracellular enzymes, including alanine aminotransferase (ALT) and aspartate aminotransferase (AST), into the bloodstream. These enzymes are therefore widely regarded as important biochemical indicators of hepatocellular injury. For example, within 48 h of acute heat stress, serum ALT and AST activities in Siberian sturgeon (*Acipenser baerii*) increased by 1.8-fold and 2.3-fold, respectively, compared with the control group, indicating substantial damage to hepatic parenchymal cells [17]. Ultrastructural observations have further confirmed the detrimental effects of high temperature on the fish liver. In blunt snout bream (*Megalobrama amblycephala*), heat exposure induced mitochondrial swelling and endoplasmic reticulum dilation in hepatocytes [18]. Similarly, rainbow trout (*Oncorhynchus mykiss*) exposed to continuing heat stress exhibited hepatic congestion and cellular degeneration, suggesting the initiation of hepatic stress responses [19].

The intestine is the primary site of nutrient absorption and barrier defense in fish and represents a critical interface between the host and the gut microbiota. Heat stress impairs intestinal function through multiple mechanisms, including disruption of intestinal mucosal architecture, damage to tight junctions, dysbiosis of the gut microbiota, and induction of inflammatory responses. Prolonged exposure to elevated temperature can result in villus atrophy, epithelial shedding, and even mucosal necrosis. Histological observations have shown that increasing stress temperature progressively reduces villus height and epithelial thickness, while significantly increasing muscular layer thickness and goblet cell abundance, indicating remodeling of the intestinal barrier. In rainbow trout, heat stress significantly elevated serum diamine oxidase (DAO) and lipopolysaccharide (LPS) levels, reflecting increased intestinal permeability and translocation of microbial products [20]. High temperature can also inhibit the synthesis and assembly of tight junction proteins (TJPs), thereby facilitating the passage of toxins and pathogens across the epithelial barrier. During acute heat exposure, both the diversity and relative abundance of intestinal microbiota are markedly altered, generally characterized by reductions in beneficial bacteria and increases in potentially harmful microorganisms. This microbial imbalance is closely associated with the host’s thermal tolerance [21]. In blunt snout bream, elevated aquaculture water temperature has also been shown to reduce appetite and feed intake, further aggravating nutritional imbalance and metabolic stress.

Gills and respiratory-related tissues are highly sensitive to temperature fluctuations because heat stress increases oxygen demand while reducing dissolved oxygen availability. Under elevated temperature conditions, fish may display respiratory distress and oxidative imbalance, accompanied by increased lipid peroxidation and altered antioxidant enzyme activities. Studies in olive flounder (*Paralichthys olivaceus*) showed that high temperature affected growth performance and antioxidant enzyme activities [22], and juvenile European seabass (*Dicentrarchus labrax*) exposed to thermal stress exhibited changes in stress indicators, oxidative biomarkers, and histopathological alterations [23]. These findings indicate that respiratory and systemic oxidative injury under heat stress should be interpreted according to species, tissue examined, exposure duration, and measured biomarkers.

Water temperature is also a key environmental factor regulating reproductive rhythms, growth and development, and life-history strategies in fish. Elevated temperature may interfere with reproductive physiology and early development, but the direction and magnitude of the response depend strongly on species, sex, developmental stage, and exposure regime. Long-term high temperature affected urogenital papilla morphology and future reproductive performance in young Nile tilapia females [3]. These findings indicate that heat stress can impair reproduction and early development, but species-specific evidence should be cited for each claim.

### 3.3. Effects of Heat Stress on the Immune and Antioxidant Systems of Fish

Heat stress exerts profound effects on the immune and antioxidant systems of fish by disrupting humoral immunity, cellular immunity, and endocrine–immune interactions. Lysozyme, which is widely distributed in the mucus, skin, gills, intestine, and serum of fish, serves as an important bacteriolytic enzyme involved in antibacterial, antiviral, and anti-inflammatory defense [24]. Under high-temperature conditions, lysozyme activity generally declines, resulting in reduced disease resistance and increased susceptibility to pathogens. Seasonal and temperature-related immune variation has been reported in Asian catfish (*Clarias batrachus*) [25], and water temperature can also affect lysozyme activity in Japanese eel (*Anguilla japonica*) [26]. Heat stress also affects the abundance and function of immune cells. Macrophages, which play central roles in innate immunity through pathogen recognition and phagocytosis, may exhibit reduced phagocytic and bactericidal activities under elevated temperatures. In addition, thermal stress can induce cellular stress responses, including increased expression of hsp70, hsp27, and xbp1 splicing, thereby altering neutrophil behavior and immune function [27]. As a potent environmental stressor, heat stress continuously activates the hypothalamic–pituitary–interrenal (HPI) axis and promotes cortisol secretion [28]. Sustained elevation of cortisol can impair immune defense through multiple mechanisms, including inhibition of lymphocyte proliferation, suppression of macrophage activity, and reduced synthesis of antibodies and lysozyme, ultimately increasing the likelihood of infectious disease outbreaks.

In addition to suppressing innate immunity, heat stress may also impair adaptive immune responses in fish [29]. Elevated temperature and prolonged cortisol exposure can inhibit lymphocyte proliferation, reduce antibody production by B cells, and alter T-cell-mediated immune regulation. These changes may weaken antigen-specific immune protection and compromise long-term immune memory or vaccine efficacy under chronic thermal stress. However, compared with innate immune responses, the effects of heat stress on B- and T-lymphocyte function in fish remain relatively poorly understood and require further investigation [30].

High water temperature not only directly induces physiological stress in fish, but also substantially alters host–pathogen interactions within aquaculture systems. Thermal stress can impair mucosal immunity, disrupt intestinal microbiota homeostasis, and weaken innate immune defenses, thereby increasing host susceptibility to opportunistic bacterial pathogens [30]. Meanwhile, many aquatic pathogenic bacteria, including *Vibrio* spp., *Aeromonas* spp., and *Edwardsiella* spp., exhibit accelerated growth, enhanced virulence factor expression, and increased transmission efficiency under elevated temperature conditions. Consequently, rising water temperatures and recurrent summer heatwaves are reshaping the epidemiological profile of bacterial diseases in aquaculture. In marine cage farming systems, particularly salmon aquaculture, extreme summer heatwaves have frequently been associated with large-scale mortality events accompanied by bacterial infections and secondary disease outbreaks. Therefore, thermal stress should be considered not only a physiological challenge, but also an important ecological driver that increases disease risk under global climate change [31].

At the oxidative stress level, elevated temperature significantly increases the production of reactive oxygen species (ROS) while simultaneously weakening antioxidant defenses, thereby inducing cytotoxic damage [32]. Heat and hypoxic stress can interact with nitric oxide-related signaling and antioxidant systems in fish, indicating that temperature-driven oxidative responses are closely linked with oxygen availability and tissue-specific metabolic regulation [33].

During the early stages of heat stress, moderate ROS accumulation may function as a signaling cue that activates antioxidant defense systems, including SOD, CAT, GPx, glutathione, and Nrf2-related pathways. However, when ROS production exceeds the compensatory capacity of these antioxidant systems, oxidative homeostasis collapses. This transition is typically accompanied by altered antioxidant enzyme activities, glutathione depletion, increased malondialdehyde (MDA) accumulation, mitochondrial dysfunction, and activation of apoptotic pathways [1,32]. Therefore, the progression from physiological adaptation to cellular injury represents a threshold-dependent process influenced by stress intensity, exposure duration, and species-specific antioxidant capacity.

### 3.4. Effects of Heat Stress on the Growth and Behavior of Fish

Water temperature directly influences fish growth efficiency by regulating a series of key physiological processes, including metabolic rate, feeding behavior, oxygen consumption, energy allocation, and digestive enzyme activity [34,35]. Different fish species possess distinct optimal thermal ranges for growth and development. Under suitable temperature conditions, fish can effectively balance energy allocation between basal metabolic maintenance and somatic growth. However, prolonged exposure to excessively high or low temperatures forces fish to divert substantial amounts of energy toward stress responses and maintenance of physiological homeostasis, thereby reducing the energy available for growth and reproduction and ultimately resulting in reduced growth performance or even growth stagnation.

Numerous studies have demonstrated that unsuitable temperature conditions can suppress fish growth, feed intake, and feed utilization efficiency. Under high-temperature stress, Japanese flounder (*P. olivaceus*) showed significant reductions in specific growth rate, feeding rate, and feed conversion efficiency [22]. Similarly, digestive enzyme activities in large yellow croaker (*L. polyactis*) were markedly decreased under elevated temperature conditions [36]. Temperature and feeding status can also influence growth-related endocrine regulation, including insulin-like growth factor signaling, although the direction of change depends on temperature regime, nutritional status, and species [37,38].

At the behavioral level, heat stress can substantially alter swimming activity, feeding behavior, and escape responses in fish. In turbot (*Scophthalmus maximus*), high-temperature exposure induced transcriptomic changes in kidney tissues, indicating systemic stress responses to elevated temperature [39]. Behavioral evidence from coastal fish species further shows that thermal tolerance differs among black rockfish (*Sebastes schlegelii*), fat greenling (*Hexagrammos otakii*), and other common species, suggesting that heat-stress effects on activity and survival are strongly species-specific [40].

### 3.5. Effects of Heat Stress on Crustaceans Such as Shrimps and Crabs

Although this review primarily focuses on fish, the responses of crustaceans to heat stress also provide valuable comparative insights, as many physiological and ecological responses are broadly conserved among aquatic ectotherms. For example, heat stress can significantly alter ecologically relevant behaviors in mantis shrimp, including *Gonodactylus childi* and *Neogonodactylus oerstedii* [41]. As water temperature increases, both species exhibit changes in respiratory behavior, as indicated by variations in pleopod movement frequency; however, the response patterns differ markedly among species and body-size classes. Smaller individuals tend to show fluctuating respiratory frequencies with increasing temperature, whereas larger individuals generally exhibit more stable or reduced respiratory activity, reflecting differences in physiological regulatory capacity. More importantly, elevated temperature strongly suppresses the frequency of their characteristic predatory and defensive “striking behavior”. Among the two species, *N. oerstedii* appears to be more thermally sensitive, with striking behavior almost completely ceasing when water temperature exceeds approximately 37.5 °C [41]. Under extreme heat conditions, some individuals also display abnormal burrow-leaving behavior, which may substantially increase their vulnerability to predation.

Heat stress also exerts pronounced effects on immunity, physiological metabolism, and intestinal microbial communities in the Chinese mitten crab (*Eriocheir sinensis*). Acute high-temperature exposure (e.g., 32 °C) can induce dynamic changes in multiple enzyme activities within the hepatopancreas and hemolymph, typically characterized by an initial increase followed by a subsequent decline. These enzymes include alkaline phosphatase (AKP), acid phosphatase (ACP), aspartate aminotransferase (AST), and lactate dehydrogenase (LDH), suggesting varying degrees of hepatopancreatic injury, reduced antioxidant capacity, and persistent physiological stress [42]. Simultaneously, glucose and total cholesterol concentrations in the hemolymph increase significantly, indicating enhanced energy metabolism and the possible activation of anaerobic metabolic pathways to meet the elevated energetic demands imposed by thermal stress.

Heat stress can additionally reshape the intestinal microbial community structure of *E. sinensis*. The relative abundances of beneficial symbiotic bacteria, such as *Candidatus Hepatoplasma* and *Marinifilum*, decrease significantly under elevated temperature conditions, whereas potentially pathogenic genera, including *Rhodococcus* and *Morganella*, become enriched [42]. Although overall microbial diversity indices may not change dramatically, the altered community composition may impair nutrient absorption and immune regulation in the host, thereby increasing the risk of disease outbreaks during high-temperature periods.

Taken together, the crustacean evidence supports the broader conclusion that elevated temperature commonly disrupts energy allocation, immune defense, behavior, and host–microbiota interactions in aquatic ectotherms (shown in Table 1). However, these responses should not be generalized directly to fish because crustaceans differ in respiratory structures, molting physiology, endocrine regulation, and microbial ecology. Therefore, these examples are used here to highlight comparative principles and to identify shared stress-response themes, while the mechanistic synthesis and applied recommendations remain centered on fish.

## 4. Molecular Regulation of Fish in Response to Heat Stress

### 4.1. The Core Regulatory Role of Heat Shock Proteins (HSPs)

Heat shock proteins (HSPs) are a group of highly conserved molecular chaperones, including both constitutive chaperone proteins and stress-induced proteins. They are upregulated under various stress conditions such as heat stress, hypoxia stress, osmotic pressure changes, ultraviolet radiation, heavy metal exposure, poisoning, protein misfolding, and microbial infection. They are key factors in maintaining cellular protein homeostasis [43]. HSPs participate in the dynamic balance of cellular protein synthesis and degradation as a whole by assisting the folding of nascent polypeptide chains, promoting the refolding or degradation of denatured proteins, and regulating protein transmembrane transport and complex assembly. In addition, HSPs are also involved in regulating processes such as cell apoptosis, immune response, and inflammatory response, and play a “hub” role in the body’s stress defense [44].

Based on molecular weight and functional characteristics, HSPs are generally classified into several major families, including HSP90, HSP70, and small heat shock proteins (sHSPs). Among these, HSP70 is the most widely distributed and extensively studied family, and its upregulation has been closely associated with enhanced host resistance to environmental stress and pathogen infection [45]. HSP70 proteins are highly conserved and are primarily involved in peptide folding, protein transmembrane transport, and regulation of the heat shock response [46]. Under stress conditions, HSP70 can suppress excessive reactive oxygen species (ROS) production, regulate death receptor signaling pathways, and modulate autophagy, thereby maintaining cellular homeostasis and exerting strong anti-apoptotic effects that are critical for cell survival and proliferation [45]. In contrast, HSP90 is more closely associated with maintaining cytoskeletal stability and preserving the conformation and activity of steroid receptors and multiple signaling proteins [47].

Numerous studies have demonstrated the involvement of HSPs in thermal stress responses in fish and crustaceans. In rainbow trout (*O. mykiss*), heat stress significantly increased the expression of HSP70 and HSP90 in intestinal tissues [48]. Under exposure to 24 °C, genes including hspa8a, hspa70a, hspa4L, and hspa5 in the liver, as well as hspa5, hspa8b, and hspa8a in the head kidney, were significantly upregulated, with hspa8a, hspa8b, and hsp70a showing the most pronounced responses, suggesting positive regulation of the Hsp70/110 family during thermal stress adaptation [49,50]. In contrast, some cold-adapted species, such as Antarctic fish, possess substantially lower upper thermal tolerance limits than temperate species. Under heat stress conditions, both inducible HSP70 and constitutively expressed HSC70 were downregulated in these species [51], indicating that organisms chronically adapted to low-temperature environments may exhibit an insufficient heat shock response when exposed to warming conditions.

Similar patterns have also been observed in other aquatic animals. In Atlantic cod (*Gadus morhua*), exposure to elevated temperature significantly induced heat-shock-responsive genes, including hsp70, in the liver, head kidney, and skeletal muscle [52]. In giant tiger prawn (*Penaeus monodon*), hsp21 expression increased markedly after heat stress, indicating its involvement in the thermal stress response [53]. However, hsp21 was downregulated after white spot syndrome virus (WSSV) infection, whereas hsp70 and hsp90 were significantly induced following *Vibrio harveyi* exposure, suggesting that HSPs may function as important mediators linking thermal stress and immune regulation [54]. Collectively, these findings indicate that HSPs represent critical molecular defense barriers that enable aquatic animals to cope with heat stress and the associated risk of pathogen invasion.

### 4.2. Antioxidant Pathway

#### 4.2.1. Nrf2-Keap1 Signaling Pathway

The Nrf2-Keap1/ARE pathway is a central signaling axis regulating cellular antioxidant and detoxification defenses. Under oxidative stress conditions, Keap1 acts as a redox-sensitive adaptor that regulates Nrf2 stability and nuclear translocation, thereby controlling the expression of antioxidant and phase II detoxification genes [55,56]. In fish exposed to heat stress, this pathway is relevant because elevated temperature commonly increases ROS production and challenges antioxidant homeostasis.

Under physiological conditions, Nrf2 binds to Kelch-like ECH-associated protein 1 (Keap1) in the cytoplasm, where it is continuously subjected to ubiquitination and proteasomal degradation through the Keap1–Cul3 E3 ubiquitin ligase complex, thereby maintaining low basal expression levels [55]. Under oxidative stress conditions, Keap1 functions as a redox-sensitive sensor. Critical cysteine residues within its intervening region (IVR), particularly Cys273 and Cys288, can be modified by ROS or electrophilic compounds, resulting in conformational changes that disrupt the activity of the Keap1–Cul3 E3 ubiquitin ligase complex and consequently inhibit Nrf2 degradation. Stabilized Nrf2 subsequently translocates into the nucleus, where it forms heterodimers with small Maf (sMaf) proteins and binds to antioxidant response elements (AREs), thereby inducing the transcription of antioxidant and phase II detoxification genes, including superoxide dismutase (SOD), catalase (CAT), glutathione peroxidase (GPx), quinone oxidoreductase 1 (NQO1), and Heme oxygenase-1 (HO-1) [56]. Through this mechanism, the Nrf2–Keap1 pathway amplifies and fine-tunes cellular responses to oxidative stress.

Under heat stress conditions, fish and other aquatic ectotherms typically experience excessive ROS production and accumulation of toxic oxidative by-products. To counteract oxidative injury, organisms rely not only on non-enzymatic antioxidants such as glutathione and ascorbic acid, but also on coordinated enzymatic defense systems involving SOD, CAT, and GPx. In Hong Kong catfish (*Clarias fuscus*), chronic heat stress altered growth, apoptosis, antioxidant enzyme activities, transcriptomic profiles, and immune-related genes [57]. These findings suggest that once the intensity or duration of heat stress exceeds the compensatory capacity of antioxidant systems, oxidative defense mechanisms may become dysregulated.

Transcriptomic analyses in Atlantic salmon (*Salmo salar*), rainbow trout (*O. mykiss*), Chinese tongue sole (*Cynoglossus semilaevis*), and snow trout (*Schizothorax richardsonii*) have demonstrated that heat stress can markedly alter the expression profiles of the Nrf2 signaling pathway and its downstream antioxidant genes [58]. In *C. fuscus*, long-term thermal stress induced hepatic injury and altered thermotolerance responses [59]. Together, these studies indicate that Nrf2-related antioxidant regulation should be discussed with attention to species, tissue, and sampling time.

#### 4.2.2. FOXO Signaling Pathway

Forkhead box O (FOXO) transcription factors play pivotal roles in cellular stress adaptation and maintenance of physiological homeostasis. Under conditions such as nutrient deprivation, oxidative stress, and genotoxic injury, FOXO proteins are activated and regulate the transcription of downstream target genes involved in stress resistance, metabolism, cell cycle control, and apoptosis [60]. In fish heat-stress studies, FOXO signaling has been particularly linked with liver injury and mitochondria-related apoptosis in Tsinling lenok trout (*Brachymystax lenok tsinlingensis*) [61,62]. These findings indicate that the FOXO pathway may function either as a pro-apoptotic or anti-apoptotic regulator depending on species-specific characteristics, stress intensity, and the stage of thermal exposure.

#### 4.2.3. PI3K/Akt Signaling Pathway

The phosphoinositide 3-kinase/protein kinase B (PI3K/Akt) signaling pathway is a highly conserved and multifunctional cellular signaling network that is widely recognized as a central regulator of cell survival and stress defense. This pathway contributes to the maintenance of physiological homeostasis through the regulation of cell survival, antioxidant responses, metabolism, and immune function. In experimental studies, increased levels of phosphorylated Akt (p-Akt) and elevated PI3K mRNA expression are commonly regarded as indicators of pathway activation. Activated Akt can interact with the Nrf2–Keap1 complex, promote the dissociation of Nrf2 from Keap1-mediated inhibition, and facilitate Nrf2 nuclear translocation, thereby inducing the expression of antioxidant genes such as SOD and CAT. Through these mechanisms, the PI3K/Akt pathway can alleviate oxidative stress and inflammatory injury and improve tissue damage, particularly in the liver.

In blunt snout bream (*Megalobrama amblycephala*), combined hypoxia and high-temperature exposure significantly affected oxidative status, immunity, and apoptosis through the PI3K/Akt signaling pathway [63]. In small abalone (*Haliotis diversicolor*), PI3K/Akt signaling was involved in hypoxia/thermal-induced immunosuppression [64]. Transcriptome analysis in Chinese tongue sole (*Cynoglossus semilaevis*) also suggested that genes associated with PI3K/Akt-related regulation may participate in early heat-stress responses [65]. These findings indicate that the regulatory effects of the PI3K/Akt pathway vary depending on species, tissue, stress intensity, and the complexity of environmental conditions.

### 4.3. Inflammation and Apoptosis Pathways

#### 4.3.1. MAPK Signaling Pathway

Mitogen-activated protein kinases (MAPKs) are members of a highly conserved family of serine/threonine protein kinases that are widely distributed across eukaryotic organisms. MAPK pathways can be activated by a variety of extracellular stimuli, including heat stress, osmotic fluctuations, reactive oxygen species (ROS), and cytokines. The classical MAPK signaling cascade consists of three sequentially activated kinase modules: MAPK kinase kinases (MAPKKKs), MAPK kinases (MAPKKs), and MAPKs, which transmit signals through stepwise phosphorylation and ultimately regulate target gene expression and cellular fate [66]. In animals, MAPK signaling is mainly composed of three major branches: extracellular signal-regulated kinase (ERK), p38 MAPK, and c-Jun N-terminal kinase (JNK). Among them, the ERK pathway is primarily associated with cell proliferation and differentiation, whereas p38 and JNK signaling are more closely involved in inflammatory responses, apoptosis, and stress adaptation. Reactive oxygen species serve as important signaling mediators in MAPK pathway activation. Excessive ROS accumulation can trigger p38/JNK activation and further interact with signaling pathways such as NF-κB to regulate inflammation and apoptotic processes [67].

The involvement of MAPK signaling in fish heat-stress responses has been demonstrated in multiple studies. In turbot (*Scophthalmus maximus*), the effects of high-temperature stress interacted with dietary lipid levels, and appropriate dietary fat supplementation suppressed excessive MAPK activation, thereby alleviating tissue damage [68]. In Nile tilapia (*Oreochromis niloticus*), heat stress activated apoptosis- and autophagy-related regulation and promoted follicular atresia [69]. In Hong Kong catfish (*Clarias fuscus*), long-term thermal stress reshaped head-kidney tolerance to acute heat shock by regulating energy metabolism and immune responses [70]. Genome-wide identification of MAPK family genes in turbot also showed that MAPK members participate in abiotic and biotic stress responses [71]. Therefore, the functional divergence, evolutionary conservation, and species-specific regulatory characteristics of MAPK signaling in fish thermal adaptation still require further systematic investigation.

#### 4.3.2. NF-κB Pathway

The nuclear factor kappa B (NF-kappa B) signaling pathway is a critical regulatory hub linking inflammation, oxidative stress, and apoptosis, and increasing attention has been paid to its role in fish stress physiology. In Tsinling lenok trout (*Brachymystax lenok tsinlingensis*), high-temperature-induced differential expression of miRNAs was associated with liver inflammatory responses [72]. General NF-kappa B studies further indicate that this pathway can regulate both cell survival and inflammation, thereby providing a mechanistic basis for interpreting heat-induced inflammatory injury [73,74].

NF-κB1, which encodes the precursor of the NF-κB p50 subunit, plays complex and context-dependent roles in apoptosis regulation. On the one hand, NF-κB activation can inhibit the transcription of pro-apoptotic genes such as Bax and Bad while promoting the expression of anti-apoptotic genes including *Bcl-2* and *XIAP*, thereby exerting cytoprotective effects. On the other hand, excessive or prolonged activation of NF-κB may enhance the production and release of pro-inflammatory cytokines, aggravating tissue injury and inflammatory responses [74].

In addition, NF-κB signaling interacts extensively with MAPK pathways such as JNK and p38, forming a coordinated “ROS–MAPK–NF-κB” regulatory network involved in thermal stress responses. Through these interactions, NF-κB plays important regulatory roles in heat stress-induced inflammation, oxidative damage, immune dysregulation, and cell death in aquatic organisms.

### 4.4. Metabolic Regulatory Pathway

#### 4.4.1. FXR/TGR5 Signaling Pathway

Farnesoid X receptor (FXR) is a bile-acid-dependent nuclear receptor that is predominantly expressed in tissues involved in enterohepatic bile acid circulation, particularly the liver and ileum. Upon activation by hydrophobic bile acids, FXR forms a heterodimer with retinoid X receptor (RXR) and binds to specific response elements within target gene promoters, thereby regulating gene transcription. In the liver, activated FXR induces the expression of small heterodimer partner (SHP), which subsequently suppresses the expression of CYP7A1 and CYP8B1, leading to reduced bile acid synthesis. In mammals, intestinal FXR activation stimulates the secretion of fibroblast growth factor 15/19 (FGF15/19), which enters the liver through the portal circulation and suppresses CYP7A1 expression, thereby forming a classical liver–intestine negative feedback loop. Although FGF19-like signaling components have been identified in several teleost species, the evolutionary conservation, regulatory mechanisms, and physiological significance of the FXR–FGF19-like axis in fish remain incompletely understood. Therefore, this pathway in fish should currently be regarded as a potentially conserved regulatory mechanism rather than a fully validated mammalian-equivalent signaling axis. In addition, FXR can regulate tight junction proteins through the FXR–MLCK pathway, enhance epithelial barrier integrity, suppress NF-κB activation, alleviate inflammatory responses, and promote MUC2 expression to strengthen the mucus barrier [75].

TGR5 is a G protein-coupled bile acid receptor that is primarily expressed in the gallbladder, ileum, colon, and other metabolically active tissues. Upon activation, TGR5 exerts its biological functions mainly through the Gαs-adenylate cyclase-cAMP-PKA signaling axis. In adipose tissues, TGR5 activation promotes the conversion of thyroxine (T4) to triiodothyronine (T3), thereby increasing energy expenditure [76]. In enteroendocrine cells, it stimulates glucagon-like peptide-1 (GLP-1) secretion and participates in glucose homeostasis [77].

In fish, the FXR/TGR5 signaling pathway is closely associated with bile acid metabolism, lipid metabolism, glucose regulation, and intestinal barrier maintenance. Studies in large yellow croaker (*Larimichthys crocea*) and mandarin fish (*Siniperca chuatsi*) have shown that dietary bile acids can regulate lipid metabolism, suppress lipid accumulation, and improve gut health, partly through activation of FXR-related signaling [78,79]. Activation of the FXR pathway can suppress the expression of lipogenic regulators such as sterol regulatory element-binding protein 1 (SREBP1), reduce lipid synthesis, and upregulate genes involved in lipolysis and fatty acid β-oxidation, thereby promoting lipid mobilization. Bile acids can also stimulate cAMP production, activate the AMPK/SREBP1 signaling pathway, and alleviate lipid accumulation induced by high-starch diets. Furthermore, bile acids may activate protein kinase B (Akt), which subsequently suppresses FOXO1 expression and downregulates glucose-6-phosphatase, thereby contributing to improved glucose metabolism [80]. At the intestinal level, activation of FXR/TGR5 signaling can inhibit NF-κB nuclear translocation, alleviate intestinal mucosal inflammation, and contribute to the maintenance of intestinal structure and barrier function under heat stress conditions.

Compared with the HSP, Nrf2-Keap1, MAPK, and NF-kappa B pathways, FXR/TGR5 signaling appears to link heat-stress mitigation more directly with nutrient sensing, lipid mobilization, glucose homeostasis, and intestinal barrier protection. This distinction is important because nutritional interventions such as bile acid supplementation may not simply provide energy substrates, but may also reshape endocrine–metabolic signaling and inflammatory tone. Nevertheless, most current evidence is derived from specific species, diets, and experimental temperatures; therefore, the protective effects of FXR/TGR5 activation should be interpreted as context-dependent rather than universally applicable across all fish or aquaculture systems.

In addition to FXR/TGR5 signaling, the Apelin/APJ pathway has recently emerged as another potential regulatory axis involved in thermal adaptation. Multi-omics analyses in zebrafish embryos have shown that multiple genes associated with the Apelin/APJ pathway participate in the regulation of developmental acceleration and energy metabolism under elevated temperature conditions [81]. These findings suggest that the Apelin/APJ pathway may contribute to the reshaping of developmental rhythms and energy allocation during thermal stress adaptation.

#### 4.4.2. PPAR Signaling Pathway

Peroxisome proliferator-activated receptors (PPARs) are an important class of ligand-activated transcription factors belonging to the nuclear receptor superfamily and are widely regarded as cellular “lipid sensors”. In addition to their central roles in lipid metabolism, PPARs are also involved in the regulation of immune responses, inflammation, vascular function, cell proliferation, and cellular differentiation [82]. In mammals, the PPAR family mainly consists of three subtypes: PPARα, PPARβ/δ, and PPARγ. Although these isoforms share similar structural features, they differ in tissue distribution, ligand preference, and biological function. Among them, PPARγ is highly expressed in adipose tissues and serves as a key regulator of adipogenesis and systemic lipid metabolism, while also being closely associated with insulin sensitivity and immune–inflammatory regulation.

The regulatory mechanism of PPARs follows a classical ligand-dependent transcriptional activation model. Upon binding to fatty acids or their metabolic derivatives, activated PPARs function as nuclear receptors and form heterodimers with retinoid X receptor (RXR). These complexes subsequently bind to peroxisome proliferator response elements (PPREs) located within the promoter regions of target genes and recruit transcriptional co-activators or co-repressors, thereby upregulating or downregulating the expression of downstream genes involved in lipid transport, fatty acid oxidation, glucose metabolism, and energy balance [83].

In turbot (*Scophthalmus maximus*), high-temperature exposure has been shown to activate PPAR-gamma signaling, which regulates fatty acid uptake and transport through upregulation of lipid transport-related genes such as fatp and cd36, thereby contributing to the maintenance of lipid metabolic homeostasis under heat stress conditions [82]. More broadly, PPARs in ray-finned fish are considered important but functionally diverse lipid-sensing nuclear receptors, and their specific roles in heat-stress adaptation require species-specific validation [84]. Under heat stress conditions, PPAR signaling may contribute to metabolic adaptation by reshaping lipid metabolic patterns, but this inference should be interpreted cautiously across species and culture conditions. The key molecular pathways related to fish heat-stress responses and their main functions are shown in Table 2.

**Figure 2:**
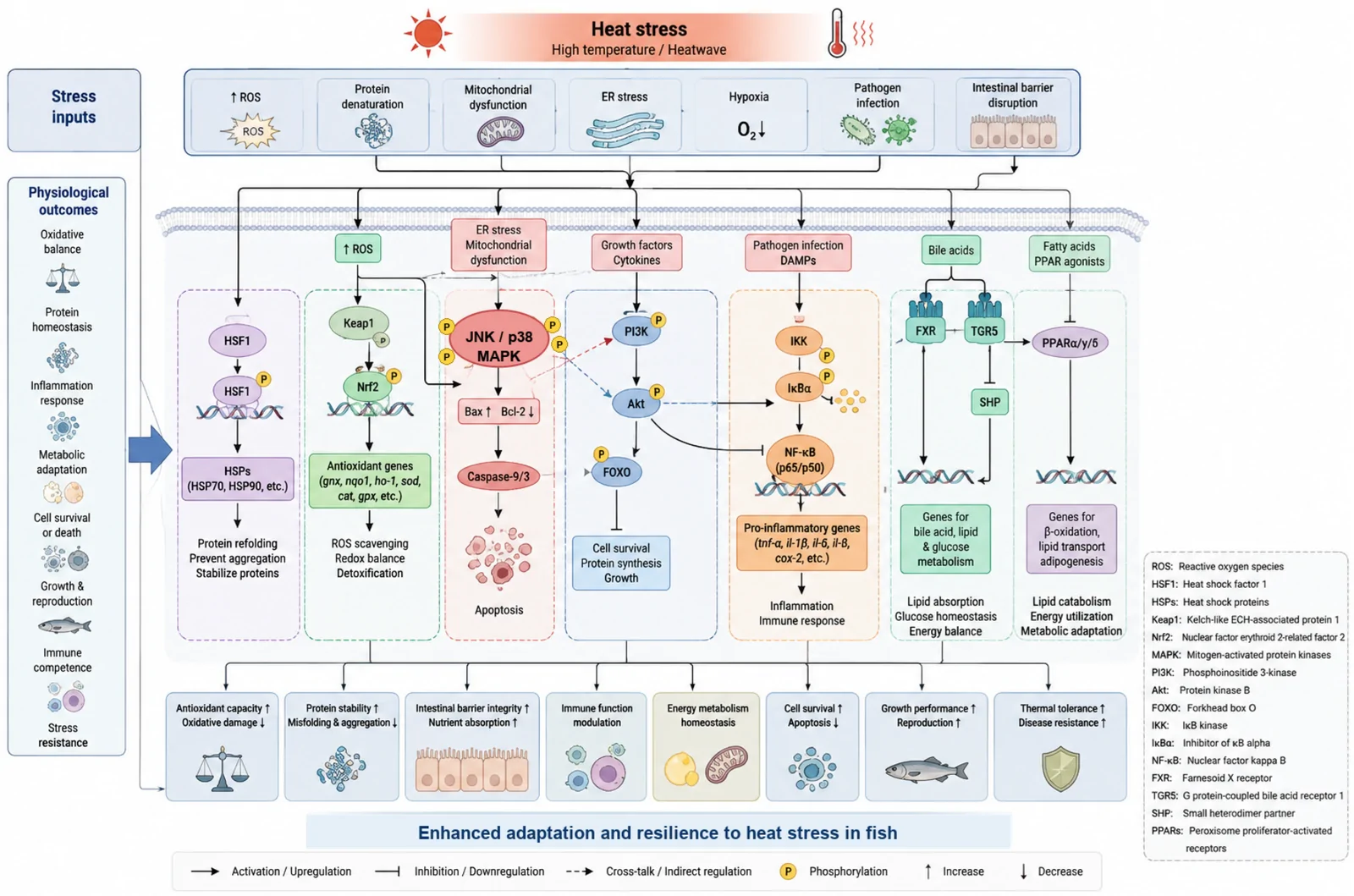
Integrated molecular signaling network involved in heat-stress responses in fish.

Taken together, these signaling pathways do not function independently but constitute an interconnected regulatory network in which oxidative stress, inflammation, apoptosis, energy metabolism, and intestinal barrier homeostasis are coordinately regulated during heat stress. Crosstalk among HSPs, Nrf2-Keap1, PI3K/Akt, MAPK, NF-κB, FOXO, FXR/TGR5, and PPAR signaling collectively determines the adaptive capacity and thermal tolerance of fish. An integrated overview of these molecular interactions is presented in Figure 2.

## 5. Adaptive Strategies of Fish in Response to Heat Stress

### 5.1. Environmental Acclimation

As poikilothermic animals, fish exhibit a certain degree of thermal plasticity. Appropriate temperature acclimation has been shown to improve thermal tolerance, which is reflected by shifts in critical thermal limits under changing environmental conditions. In two endangered fish species in China, acclimation temperature affected thermal tolerance, hypoxia tolerance, and swimming performance [85]. These findings suggest that appropriate control of warming rate and acclimation duration may improve survival and growth performance during summer high-temperature periods, but acclimation protocols should be optimized for each cultured species.

### 5.2. Nutritional Regulation

Nutritional regulation is another important strategy for alleviating the adverse effects of heat stress in fish. Rather than acting as universal solutions, functional nutrients should be selected according to species, basal diet composition, heat-stress intensity, and the physiological pathway being targeted, such as antioxidant defense, intestinal barrier integrity, immune function, or lipid metabolism.

Among functional feed additives, allicin possesses both growth-promoting and immunostimulatory properties. In striped catfish (*Pangasianodon hypophthalmus*), dietary allicin supplementation mitigated heat stress by improving growth performance, stress biomarkers, antioxidant status, and immune responses [86]. Bile acids, which function as signaling nutrients, can improve lipid and glucose metabolism through FXR/TGR5-related pathways while simultaneously supporting intestinal barrier function and antioxidant capacity. Studies in large yellow croaker (*Larimichthys crocea*), mandarin fish (*Siniperca chuatsi*), and largemouth bass (*Micropterus salmoides*) have shown that bile acids can regulate lipid metabolism, gut health, and metabolic signaling [78,79,80]. In addition, appropriate dietary lipid levels may improve thermal tolerance in some fish species; in juvenile turbot, diets containing approximately 12% lipid alleviated high-temperature-induced adverse effects on growth, lipid metabolism, antioxidant capacity, and immune responses [68]. Evidence from rainbow trout also highlights the importance of intestinal injury and microbiome–metabolome–transcriptome interactions under heat stress [48], suggesting that nutritional strategies targeting gut health may be useful but require direct validation.

Overall, adaptive strategies should be selected according to species-specific thermal tolerance, life stage, culture system, exposure duration, and the dominant physiological constraint. Short-term heatwaves may require rapid water-quality management, oxygenation, and feeding adjustment, whereas chronic seasonal warming is more likely to benefit from gradual acclimation, selective breeding, and diet formulations targeting antioxidant defense, intestinal integrity, and metabolic flexibility. Importantly, functional additives should not be treated as universal solutions; their efficacy depends on dose, basal diet composition, environmental co-stressors, and whether the targeted pathway is actually limiting under the given heat-stress scenario.

## 6. Implications for Climate-Resilient Aquaculture and Fishery Resource Conservation Under Global Warming

Under global warming, the dissolved oxygen content of the world’s oceans has declined during recent decades, and this trend is expected to continue [86]. Ocean warming and deoxygenation can compress aerobic habitat space, alter species distribution, and affect the productivity and resilience of marine fisheries [87]. Climate warming affects not only aquaculture production systems, but also commercial fisheries, recreational fisheries, and the ecological stability of rivers, lakes, wetlands, and delta ecosystems. The impacts of climate warming on fishery production and food security are complex and species-dependent. Some species, such as deep-water rose shrimp, may expand their distribution range and population density under warming and salinization conditions, resulting in temporary increases in fishery resources [88]. In contrast, many cold-water and temperate commercial fish species are facing habitat contraction, reduced reproductive success, and increased disease risk under continued warming.

At the same time, fishery resources are subjected not only to climate- and environment-related pressures, but also to long-term exploitation and habitat degradation caused by human activities. Habitat protection is essential for maintaining the ecological foundation that supports the long-term sustainability of fishery resources. Human-induced habitat degradation, including coastal overdevelopment, pollution input, ocean acidification, and marine heatwave events, is accelerating the deterioration of key ecosystems. Coral reefs, for example, support exceptionally high levels of marine biodiversity but are increasingly threatened by warming and other anthropogenic pressures [89]. Rational utilization of fishery resources cannot rely solely on individual awareness; instead, effective institutional regulations are required, including scientifically based fishing gear management, seasonal and regional fishing bans, catch quotas, and the establishment of marine protected areas.

Overall, addressing climate change is no longer merely an environmental issue associated with sustainable aquaculture and fisheries, but has become a strategic challenge closely linked to global food security and social stability. Increasingly frequent extreme thermal events are driving a future-oriented transformation of fisheries and aquaculture systems, highlighting the need to balance fish production, resource conservation, and climate adaptation. Future research should move beyond descriptive assessments of heat stress and place greater emphasis on mechanism-based intervention strategies. For example, HSP70, HSP90, Nrf2-related antioxidant genes, and immune-associated indicators may serve as potential biomarkers for thermal tolerance screening and selective breeding. In addition, nutritional strategies targeting the FXR/TGR5, PPAR, Nrf2–Keap1, and PI3K/Akt pathways may provide precise approaches for improving metabolic stability, maintaining intestinal barrier integrity, and enhancing antioxidant capacity under high-temperature conditions. The integration of multi-omics, phenomics, disease ecology, and nutritional regulation will be essential for the development of climate-resilient aquaculture systems.

Heat-stress-induced molecular and cellular damage can ultimately scale up to influence individual fitness and population stability. For instance, mitochondrial dysfunction and oxidative damage may reduce energy availability for growth and reproduction, while intestinal barrier disruption and immune suppression can increase susceptibility to disease. In addition, endocrine disturbances may impair reproductive performance. At the population level, these physiological impairments may contribute to reduced recruitment, altered age structure, increased mortality during heatwave events, and local population decline, particularly in thermally sensitive or endemic species.

From the perspective of future development, fisheries and aquaculture should gradually shift toward ecologically sustainable and climate-adaptive production systems. System resilience may be enhanced through the optimization of aquaculture models, promotion of ecological and integrated multi-trophic aquaculture, and the development of smart fisheries and digital management technologies. At the same time, greater attention should be paid to the livelihood security of small-scale fishers and coastal communities in order to achieve development goals that simultaneously emphasize low-carbon production, high efficiency, and climate adaptation. In terms of resource conservation, management strategies should move beyond single-species approaches toward ecosystem-based management. Strengthening regional and global cooperation in management and technology, expanding marine protected area networks, implementing precautionary management strategies, and improving environmental monitoring and early-warning systems will all contribute to enhancing the resilience and recovery capacity of aquatic ecosystems.

In conclusion, heat stress affects fish through an interconnected cascade that begins with environmental warming and hypoxia, progresses through metabolic reprogramming, oxidative injury, immune dysregulation, tissue damage, and behavioral change, and ultimately influences aquaculture productivity and fishery resource stability. The main practical implication is that climate-resilient aquaculture should combine early-warning systems, water-quality control, thermal acclimation, selective breeding, and mechanism-based nutritional regulation. The current knowledge base remains limited by uneven species coverage, short experimental exposure periods, inconsistent temperature regimes, and insufficient integration of laboratory mechanisms with field-scale heatwave events. Future studies should therefore prioritize long-term and multi-stressor experiments, standardized thermal-challenge protocols, cross-species comparisons, validation of molecular biomarkers, and field trials that connect omics-based mechanisms with survival, growth, reproduction, disease resistance, and production outcomes.

## Figures and Tables

**Table 1:** Main physiological effects of heat stress on fish.

Impact System	Main Changes	Representative Performance	Typical Research Species	References
Protein metabolism	Reduced protein deposition; altered amino acid metabolism; HSP-related proteostasis responses	Growth inhibition; impaired metabolic balance	*C. carpio* var. *qingtianensis*; *L. rohita*; *S. constricta*	[8,9,10]
Lipid metabolism	Enhanced lipolysis; suppressed lipid synthesis; elevated FFA; membrane lipid remodeling	Hepatic lipid-metabolism disorder; altered energy mobilization	*O. mykiss*; *S. constricta*	[11,12]
Carbohydrate metabolism	Altered blood glucose; enhanced gluconeogenesis/glycogenolysis; context-dependent glucose homeostasis	Energy-metabolism remodeling	*L. crocea*; *C. carpio* var. *qingtianensis*	[8,15]
Liver injury	Elevated ALT/AST; mitochondrial swelling; hepatic congestion or cellular degeneration	Hepatocyte injury; impaired metabolic and detoxification capacity	*A. baerii*; *M. amblycephala*; *O. mykiss*	[19,20,21]
Intestinal injury	Villus atrophy; barrier disruption; microbiota imbalance	DAO and LPS elevation; inflammation; altered microbial metabolism	*O. mykiss*	[24,25]
Respiratory and oxidative injury	Respiratory stress; altered antioxidant enzymes; lipid peroxidation; histopathological alterations	Dyspnea risk; oxidative damage; tissue injury	*P. olivaceus*; *D. labrax*	[14,26,27]
Reproduction and early development	Altered reproductive morphology; oocyte stress responses; follicular atresia	Reduced reproductive performance; developmental impairment	*O. niloticus*; *D. rerio*	[3]

**Table 2:** Key molecular pathways related to fish heat-stress responses and their main functions.

Path Name	Main Function	Changes Under Heat Stress	Representative Species	References
HSPs (HSP70/HSP90)	Protein folding; proteostasis maintenance; anti-apoptotic and immune regulation	Generally upregulated, but responses are species- and tissue-dependent	*O. mykiss*; *H. antarcticus*; *G. morhua*; *P. monodon*	[43,44,45,46,47,48,49,50,51,52,53,54]
Nrf2-Keap1	Antioxidant defense; detoxification enzyme expression	ROS accumulation may activate Nrf2-related antioxidant responses	*C. fuscus*; *C. semilaevis*; *other fish models*	[55,56,57,58,59,65]
FOXO	Cell-cycle control; stress adaptation; apoptosis regulation	Context-dependent dual regulation of apoptosis and stress adaptation	*B. lenok tsinlingensis*	[60,61,62]
PI3K/Akt	Cell survival; antioxidant response; immune regulation	Activation or suppression depends on species, tissue, and co-stressors	*M. amblycephala*; *H. diversicolor*; *C. semilaevis*	[63,64,65]
MAPK (ERK/JNK/p38)	Inflammation; apoptosis; metabolism; stress signaling	p38/JNK-related signaling is often involved in heat-stress injury	*S. maximus*; *O. niloticus*; *C. fuscus*	[68,69,70,71,72]
NF-kappa B	Immunity; inflammation; apoptosis	Inflammatory activation and context-dependent cell-survival regulation	*B. lenok tsinlingensis*	[72,73,74]
FXR/TGR5	Bile-acid signaling; lipid metabolism; glucose regulation; intestinal barrier	Lipid regulation, anti-inflammatory signaling, and barrier protection	*L. crocea*; *S. chuatsi*; *M. salmoides*	[75,76,77,78,79,80]

## Data Availability

The data that support the findings of this study are available from the corresponding author upon reasonable request.
